# A Novel Recognition by the E3 Ubiquitin Ligase of HSV-1 ICP0 Enhances the Degradation of PML Isoform I to Prevent ND10 Reformation in Late Infection

**DOI:** 10.3390/v15051070

**Published:** 2023-04-27

**Authors:** Behdokht Jan Fada, Udayan Guha, Yi Zheng, Eleazar Reward, Elie Kaadi, Ayette Dourra, Haidong Gu

**Affiliations:** Department of Biological Sciences, Wayne State University, Detroit, MI 48202, USA

**Keywords:** ICP0, ND10 formation, E3 ubiquitin ligase, SUMO-interaction, virus-host interactions

## Abstract

Upon viral entry, components of ND10 nuclear bodies converge with incoming DNA to repress viral expression. The infected cell protein 0 (ICP0) of herpes simplex virus 1 (HSV-1) contains a RING-type E3 ubiquitin ligase that targets the ND10 organizer, PML, for proteasomal degradation. Consequently, ND10 components are dispersed and viral genes are activated. Previously, we reported that ICP0 E3 differentiates two similar substrates, PML isoforms I and II, and demonstrated that SUMO-interaction has profound regulatory effects on PML II degradation. In the present study, we investigated elements that regulate the PML I degradation and found that: (i) two regions of ICP0 flanking the RING redundantly facilitate the degradation of PML I; (ii) downstream of the RING, the SUMO-interaction motif located at residues 362–364 (SIM_362–364_) targets the SUMOylated PML I in the same manner as that of PML II; (iii) upstream of the RING, the N-terminal residues 1–83 mediate PML I degradation regardless of its SUMOylation status or subcellular localization; (iv) the reposition of residues 1–83 to downstream of the RING does not affect its function in PML I degradation; and (v) the deletion of 1–83 allows the resurgence of PML I and reformation of ND10-like structures late in HSV-1 infection. Taken together, we identified a novel substrate recognition specific for PML I, by which ICP0 E3 enforces a continuous PML I degradation throughout the infection to prevent the ND10 reformation.

## 1. Introduction

Protein posttranslational modification (PTM) is a regulatory mechanism that effectively controls protein stability, activity and specificity. PTM acts rapidly and reversibly, providing a powerful tool to fine-tune the complex functionality of a living cell in an ever-changing environment. Ubiquitination is a major form of PTM that participates in many aspects of cellular pathways [1]. The process involves three sequential enzymatic reactions carried out by the E1 ubiquitin activating enzyme, E2 ubiquitin transferring enzyme and E3 ubiquitin ligase, to link a 76-residue ubiquitin to the lysine residues of a substrate protein and change the stability or activity of the substrate. Ubiquitin linked to a substrate can also be ubiquitinated to generate a variety of polyubiquitin chains. The diverse types of ubiquitin linkages and polyubiquitin chains give a protein various means to modulate its proportion, distribution, interaction, and subsequently its overall function [2,3,4]. Naturally, viruses extensively exploit the host ubiquitin system for their own advantage [4,5,6].

HSV-1 is a ubiquitous human pathogen that infects more than 70% of the adult population and causes a wide array of clinical manifestations ranging from mild sores to severe encephalitis. Upon HSV-1 infection, the incoming viral genome must encounter waves of host anti-viral defenses while the virus attempts to establish efficient transcription/replication. One of the major anti-viral responses comes from nuclear domain 10 (ND10s). ND10s, also known as promyelocytic leukemia nuclear bodies (PML-NBs), are dynamic nuclear structures involved in numerous cellular functions, such as gene regulation, cell-cycle regulation, apoptosis, and antiviral defenses [7,8,9]. To date, more than 150 proteins have been identified as ND10 components. Among them, some are permanent residents, such as promyelocytic leukemia (PML) protein and speckled protein 100 kDa (Sp100), while many others are transient components recruited to ND10 upon stimulations [9,10]. The anti-viral effects of ND10 are reflected in the facts that the size and number of ND10 increase with interferon treatment but diminish in virus infections [11,12].

ICP0 is a multifunctional immediate early protein critical for HSV-1 replication at low multiplicity of infection in cultured cells. It promotes downstream viral expression through combating host restrictive factors on multiple fronts, such as interferon (IFN) responses [13,14,15,16], chromatin repression, and DNA damage responses [17,18,19,20,21]. Some of the counter-antiviral activities of ICP0 rely on the dynamic interaction between ICP0 and ND10 [22].

Many ICP0 functions are carried out via an E3 ubiquitin ligase activity located in the RING finger domain of ICP0. Using the RING-type E3 ubiquitin ligase, ICP0 triggers various host proteins such as the ND10 organizers, PML and Sp100, to undergo ubiquitin-mediated proteasomal degradation [23]. Upon HSV-1 entry, ND10 components converge to the incoming DNA to restrict viral expression [7,24,25]. ICP0 E3-mediated PML and Sp100 degradation help to disperse ND10 components and thereby release the DNA repression imposed by ND10 [26,27]. The siRNA knockdown of PML and Sp100 enhances the growth of HSV-1 in the absence of ICP0 [28,29].

ICP0 also modulates cellular pathways via protein–protein interactions. For example, ICP0 interacts with CoREST to dislodge histone deacetylases (HDACs) from the REST/CoREST/HDACs complex and therefore modifies the acetylation status of histone and activates viral gene expression [17,30,31]. Interestingly, mutations in the CoREST binding site located in the distal C-terminus of ICP0 can influence the E3 ubiquitin ligase activity located in the N-terminal RING domain [30], suggesting that the ICP0 E3 ubiquitin ligase activity cooperates with the ICP0-CoREST interaction during the HSV-1 infection. With the complexity and intricacy of the cellular ubiquitin system, the cooperation between the E3 ubiquitin ligase and the interactive domains of ICP0 may empower HSV-1 to fine-tune the activity and/or specificity of ICP0 E3 to achieve a concerted infection. Although the ICP0 E3 ubiquitin ligase activity has been known for two decades [32,33] and new proteins are being continuously added to the list of ICP0 substrates [34,35], the regulatory mechanisms governing the activity and specificity of ICP0 E3 remain unclear. This lack of knowledge hinders the understanding of early maneuvers between incoming HSV-1 and host defenses, which determine the ultimate outcomes of HSV-1 infection.

In preceding studies, we reported that a SUMO-interaction motif (SIM) located at ICP0 residues 362–364 (SIM_362–364_) is required for ICP0-mediated degradation of PML isoforms II, IV and VI, but not PML I [36]. Using PML II as the model substrate, we have thoroughly characterized the effects of SUMO-SIM interactions on the SIM_362–364_-dependent substrate recognition in HSV-1 infection [37]. In the present study, we focus on delineating the regulations of the SIM_362–364_-independent degradation of PML isoform I triggered by ICP0 E3. We found that a bipartite PML I-interaction domain composed of sequences both upstream and downstream of the RING [36] redundantly facilitates the ubiquitination and degradation of PML I. We report that SIM_362–364_ is the essential element downstream of the RING that recognizes and degrades the SUMOylated PML I, same as that in the recognition of PML II, VI, and IV. Moreover, ICP0 uses the N-terminal sequences upstream of RING to additionally target PML I regardless of its SUMOylation status and subcellular localization. Without the N-terminus, the PML I protein reappears in late infection to form ND10-like structures in the nucleus. Likely, this novel ubiquitination mechanism carried out by the ICP0 N-terminus ensures a continuous degradation of PML I to prevent the reformation of ND10 throughout the HSV-1 infection cycle.

## 2. Materials and Methods

### 2.1. Cells and Viruses

HEp-2 TetOn cells were cultured in DMEM medium (Invitrogen, Waltham, MA, USA) supplemented with 10% FBS and 6 μg/mL blasticidin (Invitrogen). HEp-2 TetOn cells stably expressing PML I were cultured in DMEM supplemented with 10% FBS, 6 μg/mL blasticidin and 100 μg/mL zeocin (Invitrogen), as previously described [36]. Recombinant viruses RHG101, RHG105, RHG110, RHG118, RHG120, and RHG130 have been described elsewhere [22,38].

### 2.2. Construction of Recombinant HSV-1

The XhoI-MluI fragments from plasmids pHG121, pHG124, pHG136, and RHG114, which contain ICP0 mutants lacking residues 242–341, residues 242–391, residues 242–341 and 393–441, and residues 393–441, respectively [38], were used to replace the central region of ICP0 in the plasmid pHG105, in which the ICP0 residues 1–83 had been deleted [22]. These subcloning procedures generated the double deletion plasmids pHG165, pHG167, pHG168 and pHG169.

The NruI-MluI fragments from plasmids pHG123 and pHG130, which contain ICP0 mutants lacking residues 343–441 and bearing I362G/V363A/I364G substitutions in SIM_362–364_, respectively [36,38], were used to replace the central region of ICP0 in pHG105 and generated the double deletion plasmids pHG166 and pHG170.

In a two-step jump strand PCR, the upstream primer pair 5′-GACGACGACCTGGACGACGCAGACTACGTACCGC-3′ and 5′-GGCGCCGGAGGGGGCGGCGCCGCGGGAGGGCATGGGCGCCGCGGGGGGCCTGTGGGGAGAG-3′ and the downstream primer pair 5′-CTCTCCCCACAGGCCCCCCGCGGCGCCCATGCCCTCCCGCGGCGCCGCCCCCT CCGGCGCC-3′ and 5′-GGGACGCGTGGACTGGGGGGAGGGGTTTTCCTGGC-3′ were used in the first round of PCR to amplify the upstream and downstream fragments that overlap at the deletion junction. These two fragments were then mixed together to serve as the template in the second round of PCR with the same upstream forward primer and downstream reverse primer. The PCR product was digested with SnaBI and MluI to replace the central region of ICP0 in pHG105 and generated the double deletion plasmid pHG171.

For construction of plasmid pHG172, a three-step jump strand PCR was employed. In the first round of PCR, the N-terminus forward primer 5′-CCACAGGCCCCCCGCGGCGCCCATGGAGCCCCGCCCCGGAGCGAGTACC-3′ and N-terminus reverse primer 5′-CGGAGGGGGCGGCGCCGCGGGAGGGCCCCCCATCCACGCCCTGCGGCCCCA-3′ were used to PCR amplify ICP0 N-terminal residues 1–83 with overhang sequences on both ends that overlap with upstream and downstream sequences of the deletion junction in plasmid pHG171, whereas the upstream primer pair 5′-CCAGTCGCGAGCCGCGGCGC-3′ and 5′-GGTACTCGCTCCGGGGCGGGGCTCCATGGGCGCCGCGGGGGGCCTGTGG-3′ and the downstream primer pair 5′-TGGGGCCGCAGGGCGTGGATGGGGGGC CCTCCCGCGGCGCCGCCCCCTCCG-3′ and 5′-GGGACGCGTGGACTGGGGGGAGG-3′ were used to amplify both upstream and downstream fragments of the deletion junction of pHG171 with overhang sequences that overlap with the two ends of ICP0 N-terminal residues 1–83. In the second round of PCR, the N-terminus fragment was mixed with the upstream or downstream fragment of the deletion junction to be amplified by the above upstream forward primer and N-terminus reverse primer, or the N-terminus forward primer and downstream reverse primer, respectively. The two PCR products of the second round PCR were then mixed together to be amplified in the third round of PCR by the same upstream forward primer and downstream reverse primer. The final PCR product was digested with NruI and MluI to replace the central region of ICP0 in pHG105 and generated the triple mutation plasmid pHG172.

For the construction of pHG191 and pHG192, a two-step jump strand PCR was performed. In the first round, the upstream primer pair 5’-GCAGGATCCGCAGACTACGTA CCGCCCGC-3’ and 5’-CTGCGCCTGCGCCGGCTGCACCGCCGCCTCCTGCTCGAC-3’ and the downstream primer pair 5’-GTGCAGCCGGCGCAGGCGCAGGGGTCGGGCCA GGAAAAC-3’ and 5’-GCAGTCGACTTACCCGGGCCCACCCTGGCCGCG-3′ were used to amplify the upstream and downstream fragments that contain the R501Q/R503A /K504Q/R505A/R506Q substitutions (NLSmt) in ICP0, and the upstream primer pair 5’-GCAGGATCCGCAGACTACGTACCGCCCGC-3’ and 5’-TACCTTTCTCTTCTTTTTTGG CGCCGCCTCCTGCTCGAC-3′ and the downstream primer pair 5’-CCAAAAAAGAAGAGAAAGGTAGGGTCGGGCCAGGAAAAC-3’ and 5’-GCAGTCGACTTACCCGGGCCCACCCTGGCCGCG-3’ were used to amplify the upstream and downstream fragments that contain SV40 NLS (NLS_SV40_) at residues 501–506 of ICP0. The two respective fragments for introducing the NLSmt or NLS_SV40_ mutant were then mixed together to serve as the template in the second round of PCR with the same upstream forward primer and downstream reverse primer. The second round PCR products were digested with NruI and MluI to replace the NruI-MluI fragment of ICP0 in the wild type ICP0 containing plasmid pHG101 [22] and generated the plasmids pHG191 and pHG192.

**Figure 1:**
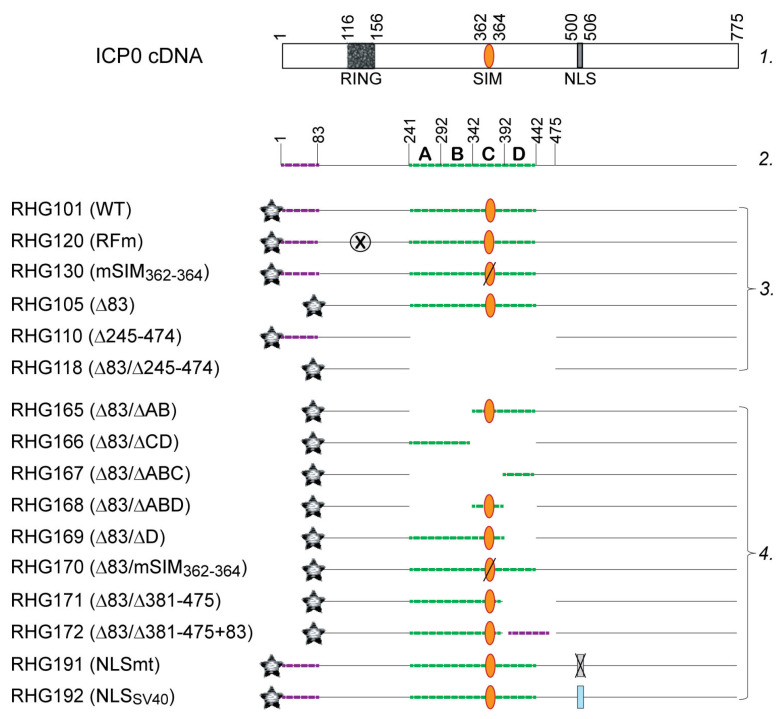
Schematic diagram of ICP0 structure of the recombinant viruses used in this study. Line 1: ICP0 cDNA with the illustration of RING-type E3 domain, SIM_362–364_ and nuclear localization sequence (NLS). Line 2: Bipartite PML I-interaction domain, with left arm labeled by purple dashed line and right arm by green dashed line. The right arm is divided into segments A–D with their residue numbers labeled on top. Group 3: Previously published recombinant viruses. Group 4: recombinant viruses constructed in this study. The gray star represents the mCherry tag fused to the N-terminus of all ICP0 constructs; the orange oval represents ICP0 SIM_362–364_, with a slash across the oval representing mutated SIM_362–364_; the crossed-out circle represents the mutated RING and the crossed-out rectangle represents the mutated NLS; the blue rectangle represents the NLS of SV40; and the absence of a line represents a deletion in the ICP0 sequence.

All the above ICP0 mutants were then cloned into pKO5 and electroporated into the *E. coli* RR1 strain that harbors ICP0-null-BAC (bacterial artificial chromosome) as described elsewhere [30]. The BAC DNAs extracted from positive colonies were transfected into U2OS cells to generate recombinant viruses. Single plaques were purified at least three times to select for the recombinant virus series from RHG165 to RHG192 (Figure 1, group 4). The presence of two copies of mutant ICP0 in both the terminal and internal repeats of the HSV-1 genome was verified by Southern blotting and sequencing.

### 2.3. Construction of HEp-2 TetOn Cell Lines Stably Expressing PML I Mutants

We previously constructed a pcDNA4/TO/HisMyc-PML II K/SIMmt plasmid, in which we introduced mutations into the three SUMOylation sites and the SIM site of a His- and Myc-tagged PML II isoform via four rounds of site-directed mutagenesis [37]. To construct the K/SIMmt version of PML I, the EcoRV-MluI fragment from an intermediate PML II plasmid containing the K65R/K160R/K490R substitutions was used to replace the EcoRV-MluI fragment of the pcDNA4/TO/HisMyc-PML I plasmid [36]. The resulting plasmid was subjected to the QuikChange II site-directed mutagenesis kit (Agilent Technologies, Santa Clara, CA, USA) to introduce the V556A/V557A/V558A/I559S substitutions with the primer pair 5′-GAGGCAGAGGAACGCGCTGCGGCGAGCAGCAGCTCGGAAGAC-3′ and 5′-GTCTTCCGAGCTGCTGCTCGCCGCAGCGCGTTCCTCTGCCTC-3′. The final plasmid was named pcDNA4/TO/HisMyc-PML I K/SIMmt. The same mutant was also introduced into the wild type PML I to generate the pcDNA/TO/HisMyc-PML I SIMmt plasmid.

To generate the L73E substitution, the plasmids pcDNA4/TO/HisMyc-PML I were used as a PCR template, and the upstream primer pair 5′-GGGGATATCGAGCCTGCACCCGCCCGA-3′ and 5′-CATCCTGAGCACAGCGTGTGCTCACAAGGCAGCAGCTTCGG-3′ and the downstream primer pair 5′-CCGAAGCTGCTGCCTTGTGAGCACACGCTGTGCTCAGGATG-3′ and 5′-CACTTCATCCTCTGCACCAGCGC-3′ were used to PCR amplify two overlapping fragments containing the L73E substitution. The two fragments were then mixed together to serve as the template for a second round of PCR by the upstream forward primer and the downstream reverse primer. The final PCR product was digested by EcoRV and KpnI to replace the EcoRV-KpnI fragment in the plasmid pcDNA4/TO/HisMyc-PML I to generate the plasmid pcDNA4/TO/HisMyc-PML I L73E.

To construct eCFP-tagged PML I, an eCFP fragment generated by primers 5′-GCAGCTAGCATGGTGAGCAAGGGCGAGGAG-3′ and 5′-GCTGATATCCTTGTACA GCTCGTCCATGC-3′ was digested by NheI and EcoRV to replace the Myc tag in pcDNA4/TO/HisMyc-PML I. The resulting plasmid was named pcDNA4/TO/eCFP-PML I.

The above plasmids were individually transfected into HEp-2 TetOn cells by the Lipofectamine 3000 transfection reagent (Invitrogen) and selected in a growth medium containing 6 μg/mL of blasticidin and 100 μg/mL of zeocin. Stable cell lines were then identified by Western blotting or fluorescent microscope after 24 h of doxycycline (Dox) induction.

### 2.4. TetOn-Based Protein Half-Life Assay

HEp-2-TetOn cells expressing a PML isoform were seeded in 35 mm plates overnight before induction with 1 μg/mL Dox for 24 h. The induced cells were incubated with a recombinant virus at 10 pfu/cell for 1 h. The inocula were removed and cells were incubated in a growth medium for 1 h before adding 100 μg/mL cycloheximide (CHX). Samples were collected at 0, 2, 4, and 6 h after the CHX treatment. The PML and ICP0 levels were detected by Western blotting to analyze the protein half-lives.

### 2.5. PML I In Vivo Ubiquitination Assay

HEp2-TetOn cells expressing HisMycPML I were mock induced or induced with 1 μg/mL Dox for 24 h, with or without the transfection of plasmids that contain either wild type or mutant ubiquitin tagged with HA in the N-terminus. The cells were then exposed to 5 pfu/cell of a recombinant virus. At 3 h post infection (hpi), cells were harvested, washed, and lysed in a UREA-lysis buffer (8 M urea, 100 mM Na_2_HPO_4_/NaH_2_PO_4_ [pH 8.0], 10 mM Tris-HCl [pH 8.0], 5 mM imidazole, 50 µM deubiquitinase inhibitor PR619 (Selleckchem, Houston, TX, USA)). Then the cell lysates were subject to brief sonication before spinning down at 14,000 rpm for 5 min. The supernatant was incubated with Ni-NTA agarose beads (Qiagen) for 3 h at room temperature with gentle agitation. The beads were then washed four times, with the UREA-lysis buffer, a UREA-wash buffer (8 M urea, 100 mM Na_2_HPO_4_/NaH_2_PO_4_, 10mM Tris-HCl [pH 6.8], 5 mM imidazole, 10 mM β-mercaptoethonal), a UREA-wash buffer containing 0.1% Triton X-100, and PBS, respectively, for 5 min each. The HisMyc PML I protein was then eluted with an elution buffer (0.2 M imidazole, 0.15 M Tris-HCl [pH 6.8], 30% Glycerol, 0.72M β-mercaptoethonal, 5% SDS). The eluates were electrophoretically separated by 7% SDS-PAGE and probed with anti-ubiquitin or anti-HA antibody by Western blotting.

### 2.6. Western Blotting

Total cell lysates in a radioimmunoprecipitation assay (RIPA) buffer (50 mM Tris [pH 7.4], 150 mM NaCl, 1 mM EDTA, 0.1% SDS, 1% NP-40, 0.25% sodium deoxycholate, 1 mM phenylmethylsulfonyl fluoride) or the pull-down precipitates described above were electrophoretically separated on SDS-PAGE, and then transferred onto a polyvinylidene difluoride (PVDF) membrane (Thermo Scientific, Rockford, IL, USA). The membrane was blocked with 1× Tris-buffered saline–Tween (TBST) (20 mM Tris [pH 7.5], 150 mM NaCl, 0.5% Tween 20) containing 5% nonfat dry milk and probed with primary antibodies as indicated in Section 3. The membrane was rinsed with TBST and incubated with horseradish peroxidase-conjugated goat anti-mouse or goat anti-rabbit secondary antibody (Sigma, St. Louis, MO, USA), rinsed, and then visualized with an ECL detection reagent (Thermo Scientific).

### 2.7. Confocal Microscopy

HEp-2 TetOn cells expressing a PML I mutant were seeded to four-well glass slides (Electron Microscopy Sciences, Hatfield, PA, USA) and mock induced or induced by 1 μg/mL of Dox before infection. For staining, cells with or without infection were fixed with 4% paraformaldehyde, permeabilized with 0.2% Triton X-100, and blocked in PBS containing 5% horse serum and 1% bovine serum albumin. Cells were then reacted with primary and secondary antibodies and slides were mounted with VectaShield (Vector Laboratories, Inc., Newark, CA, USA). Images were taken with a Leica TCS SP8 confocal microscope.

### 2.8. One-Step Viral Growth Curve

Triplicated HEp-2 TetOn cells expressing PML I were mock induced or induced by 1 μg/mL of Dox for 18 h. The cells were then infected with RHG101 or RHG105 at 0.05 pfu/cell and harvested at the indicated time points. The cell pellets were suspended in milk, sonicated, and serially diluted to titrate on U2OS cells. Viral yields were plotted with Microsoft Excel (v2016).

### 2.9. Plasmids

Plasmids pRK5-HA-Ubiquitin-WT, pRK5-HA-Ubiquitin-K48, and pRK5-HA-Ubiquitin-K63 were purchased from Addgene (Watertown, MA, USA). Plasmid pRK5-HA-Ubiquitin-K48R/K63R was a generous gift from Dr. Guang-Chao Chen (Institute of Biological Chemistry, Academia Sinica, Taipei, Taiwan) [39].

### 2.10. Antibodies

The monoclonal anti-Myc antibody was purchased from Santa Cruz Biotechnology Inc. Both polyclonal and monoclonal anti-mCherry antibodies were purchased from Clontech (Mountain View, CA, USA). The monoclonal anti-eCFP antibody was purchased from OriGene (Rockville, MD, USA). Polyclonal anti-Sp100 and anti-ATRX antibodies were purchased from Sigma. Polyclonal anti-ubiquitin and anti-SUMO2/3 antibodies were purchased from Abcam (London, UK). The high affinity anti-HA-Peroxidase was purchased from Roche. The monoclonal anti-actin antibody was purchased from Sigma. Monoclonal anti-gC, anti-ICP27, and anti-VP5 antibodies were purchased from Virusys. The rabbit polyclonal anti-ICP0 antibody was generated in this lab [40]. Horseradish peroxidase conjugated goat anti-rabbit and goat anti-mouse antibodies were purchased from Sigma. The FITC-conjugated goat anti-rabbit antibody was purchased from Sigma. The Texas Red-conjugated goat anti-mouse antibody, Alexa 488-conjugated goat anti-mouse antibody and Alexa 594-conjugated goat anti-rabbit antibody were purchased from Invitrogen.

## 3. Results

### 3.1. The Two Arms of the Bipartite PML I-Interaction Domain Redundantly Ubiquitinate PML I for Its Degradation

To study the HSV-1 elements that regulate the E3 ubiquitin ligase activity of ICP0, we manipulate HSV-1 genes and then measure the half-life of an ICP0 substrate during the infection [36,37]. Using a substrate half-life assay, we have reported that SIM_362–364_ is required for the proteasomal degradation of PML isoforms II, IV and VI, but not for PML I [36], and characterized the regulatory effects of SUMO-interaction on ICP0 E3 by using PML II as a model substrate [37]. For the SIM_362–364_-independent PML I degradation, we previously reported that two regions of ICP0, the N-terminal residues 1–83 and central residues 245–474 that flank the RING finger, namely the left and right arms of a bipartite PML I-interaction domain, redundantly interact with PML isoform I. Deletion of either region did not block ICP0 from interacting with PML I, whereas deletion of both regions completely abolished the interaction [36].

**Figure 2:**
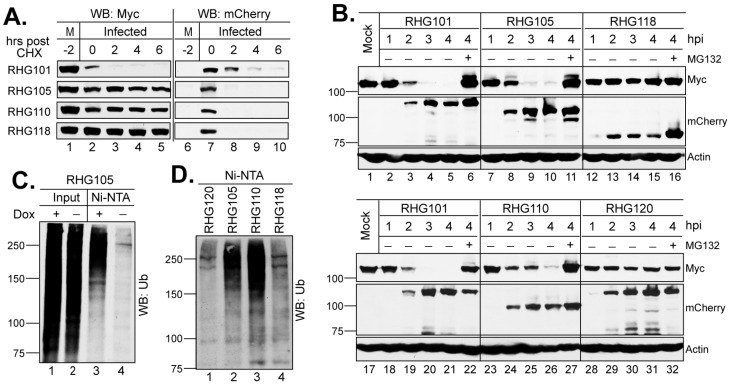
Two arms of the bipartite PML I-interaction domain redundantly target PML I for ubiquitination and degradation. (**A**) Half-life assay to detect PML I and ICP0 stability in cells infected by the indicated viruses. HEp-2 TetOn PML I cells were induced overnight and then infected by 10 pfu/cell of the indicated viruses. After 2 h of infection, CHX was added and a time course was taken. The HisMyc-tagged PML I and mCherry-tagged ICP0 were detected by the anti-Myc and anti-mCherry antibodies via Western blotting. (**B**) PML I degradation in early infection. HEp-2 TetOn PML I cells were infected with the indicated viruses in the absence or presence of MG132. At the indicated hpi, infected cell lysates were harvested to detect PML I, ICP0, or actin level by the anti-Myc, anti-mCherry, or anti-actin antibody on Western blots. (**C**) HEp-2 TetOn PML I cells were mock induced or induced with Dox, infected with RHG105, and subjected to His-tag pulldown by Ni-NTA beads under the denaturing condition. The PML I precipitates were then probed with the anti-ubiquitin antibody on Western blots. (**D**) HEp-2 TetOn PML I cells induced with Dox overnight were infected with the indicated viruses for 3 h and then subjected to in vivo ubiquitination assay as described in (**C**).

When examining the role of the bipartite PML I-interaction in the degradation of PML I we noticed that ICP0 lacking one or two arms of the bipartite domain, as shown in recombinant viruses RHG105, RHG110, and RHG118 (Figure 1), are extremely unstable after the CHX treatment in a substrate half-life assay (Figure 2A, lanes 7–10) [36]. The rapid loss of mutant ICP0 before it acts on PML I renders the PML I half-life inaccurate to reflect the regulated ICP0 E3 activity regarding PML I. Therefore, we avoided using CHX and monitored the continuous disappearance and the ubiquitination status of PML I in early infection. Results in Figure 2B showed that in cells infected by the RHG101 virus containing a wild type ICP0, PML I was quickly degraded via the proteasome pathway (Figure 2B, lanes 2–6 and 18–22), whereas the RHG120 virus that contains the C116G/C156A substitutions in RING (RFm) failed to degrade PML I (Figure 2B, lanes 28–32). While ICP0 lacking either arm of the bipartite PML I-interaction domain in the RHG105 or RHG110 virus had the ability to degrade PML I (Figure 2B, lanes 7–11 and 23–27), ICP0 missing both arms in virus RHG118 lost its ability to target PML I for degradation (Figure 2B, lanes 12–16). These results indicated that the redundant interactions between PML I and the bipartite domain flanking the RING lead to the redundancy in PML I recognition and degradation by the ICP0 E3. Either arm of the bipartite domain is sufficient but not necessary. Only when both arms are absent from interacting with PML I can PML I degradation be abolished in HSV-1 infection. To further confirm that both arms of the bipartite domain cause PML I degradation via the ubiquitination-proteasome pathway, we established an in vivo ubiquitination assay to visualize the ubiquitination of PML I catalyzed by ICP0. The HEp-2 TetOn PML stable cell lines expressing individual PML isoforms contain not only a Myc-tag but also a 6xHis-tag fused in frame to each PML isoform, as previously reported [36,37]. Under the denaturing condition, the PML I induced by Dox can be specifically pulled down by Ni-NTA beads to visualize its ubiquitination status by the anti-Ub antibody (Figure 2C, lanes 3–4). Figure 2D shows that the ICP0 lacking one arm of the bipartite domain was able to catalyze the polyubiquitination of PML I (lanes 2–3), whereas the RFm mutant and ICP0 lacking both arms failed to do so (lanes 1 and 4). These results are well correlated to the degradation of PML I in HSV-1 infection, confirming that the two arms of the bipartite PML I-interaction domain redundantly promote the degradation of PML I via polyubiquitination.

### 3.2. SIM_362–364_ Is Required for the Right Arm to Mediate the Ubiquitination and Degradation of PML I

**Figure 3:**
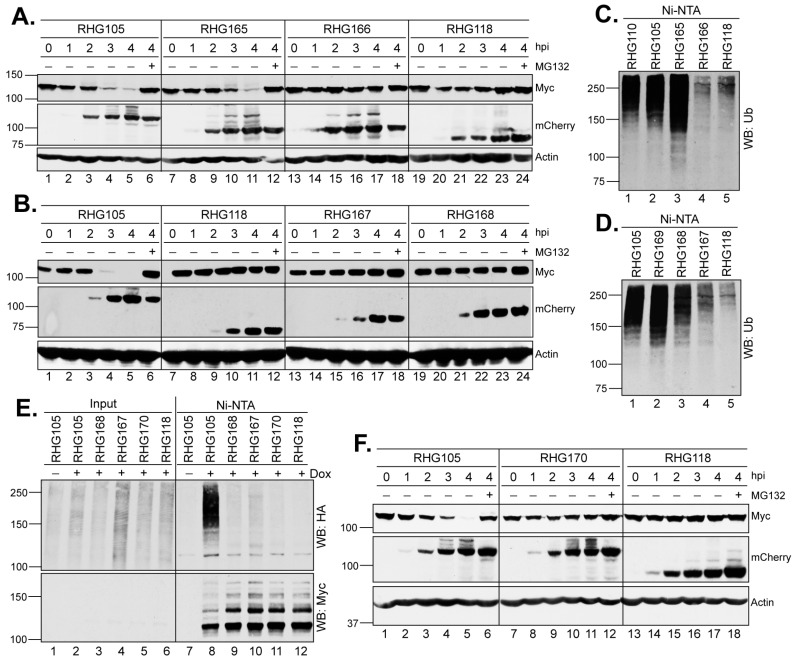
In the right arm of the bipartite PML I-interaction domain, SIM_362–364_ is required but not sufficient for the ubiquitination and degradation of PML I. (**A**,**B**,**F**) HEp-2 TetOn PML I cells were induced by Dox overnight and then infected by the indicated viruses in the absence or presence of MG132. At the indicated hpi, infected cell lysates were harvested and probed for the PML I, ICP0, and actin levels by anti-Myc, anti-mCherry, and anti-actin antibodies as described above. (**C**,**D**) HEp-2 TetOn PML I cells infected with the indicated viruses were subjected to an in vivo ubiquitination assay as described above. (**E**) HEp-2 TetOn PML I cells were transfected with HA-ubiquitin and induced by Dox for 24 h before infection with the indicated viruses. At 3 hpi, the cell lysates were subjected to His-tag pulldown and precipitates were probed for ubiquitination by an anti-HA antibody or PML I by the anti-Myc antibody.

To understand how the bipartite PML I-interaction domain facilitates the substrate recognition in PML I ubiquitination, we first conducted deletion mapping of the right arm in the absence of the left arm. Using RHG105 as the parental virus, we divided the right arm into four 50-amino-acid segments, A, B, C and D (Figure 1, line 2) and constructed recombinant viruses RHG165-168 (Figure 1, group 4). The results showed that the ICP0 containing the segment C-D in virus RHG165, but not the A-B segment in RHG166, had the ability to degrade PML I (Figure 3A). Consistent with this observation, an in vivo ubiquitination assay of these two viruses showed that PML I was successfully ubiquitinated in RHG165-infected cells but not in RHG166-infected cells (Figure 3C, lanes 3–4). Interestingly, further mapping showed that neither segment C nor D alone was sufficient to degrade PML I in the RHG168- or RHG167-infected cells (Figure 3B, lanes 13–24), or to efficiently ubiquitinate PML I (Figure 3D, lanes 3–4). To further determine whether segment C alone maintains some capability in mediating the ubiquitination of PML I, we overexpressed an HA-tagged ubiquitin (HA-Ub) to enhance the sensitivity of the ubiquitination assay. The results showed that under a denaturing condition, the poly(HA-Ub) chain was specifically pulled down by the HisMyc-PML I in RHG105-infected cells, but neither segment C nor D alone could use HA-Ub to ubiquitinate PML I (Figure 3E, lanes 8–10). Interestingly, when segment C was put together with the nonfunctional A-B segment in virus RHG169, PML I ubiquitination was fully restored (Figure 3D, lane 2), suggesting that segment C has a key motif essential for the right arm to ubiquitinate PML I, but this functional motif needs structural support from surrounding sequences, either from segment A-B or segment D. Without sufficient structural support, segment C alone in the right arm is not fully functional in carrying out the ubiquitination and degradation of PML I early in HSV-1 infection.

SIM_362–364_ is located within segment C, of which the I362G/V363A/I364G substitutions (mSIM_362–364_) are known to completely abolish the degradation of PML isoforms II, VI and IV, but not PML I in HSV-1 infection [36,37]. To examine whether SIM_362–364_ plays a role in the PML I recognition by the right arm of the bipartite PML I-interaction domain, we constructed virus RHG170, in which we deleted the left arm to remove redundancy and introduced I362G/V363A/I364G substitutions in the right arm (Figure 1, group 4). We found that the double mutant virus RHG170 lost its ability to ubiquitinate or degrade PML I (Figure 3E, lane 11, and Figure 3F, lanes 7–12). We conclude that SIM_362–364_ is the functional motif in the right arm that is required for the ubiquitination and degradation of PML I in HSV-1 infection.

### 3.3. The Right Arm of the Bipartite PML I-Interaction Domain Recognizes Only the SUMOylated PML I, While the Left Arm Mediates PML I Degradation Independent of SUMO-SIM Interaction

**Figure 4:**
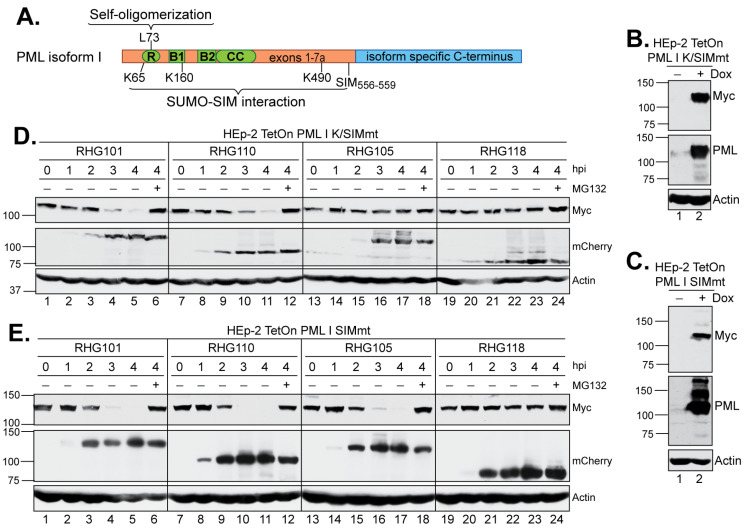
Effects of mutations in the SIM and/or SUMOylation sites of PML I on its degradation by the left or right arm of the bipartite PML I-interaction domain. (**A**) cDNA structure of PML isoforms, with the RING-BOX-Coiled Coil (RBCC) domain in shared N-terminus in green and the varied C-terminus in blue. Residues responsible for self-oligomerization (L73), SUMOylation (K65, K160 and K490) and SIM (556-559) are illustrated. (**B**,**C**) Expression level of the K/SIMmt (**B**) and SIMmt (**C**) mutants of PML I in the absence or presence of Dox induction. (**D**,**E**) HEp-2 TetOn PML I K/SIMmt cells (**D**) and HEp-2 TetOn PML I SIMmt cells (**E**) were induced overnight and infected by the indicated viruses. At the indicated hpi, infected cell lysates were harvested and protein levels of PML I mutants, ICP0, and actin were probed as described above.

In the shared PML N-terminus, the residues K65, K160 and K490 are the sites of PML SUMOylation, whereas one SUMO-interaction motif is located in residues 556–559 (Figure 4A) [41]. To further understand the role of SUMO-SIM interactions in the PML I degradation mediated by ICP0 E3, we constructed two PML I mutants. In the first one, named PML I K/SIMmt, we introduced the K65R/K160R/K490R substitutions in the three major SUMOylation sites and the V556A/V557A/V558A/I559S substitutions in the SIM site [41]. In the second one, named PML I SIMmt, we maintained the SUMOylation sites but introduced the V556A/V557A/V558A/I559S substitutions in the SIM site (Figure 4A).

HEp2-TetOn cell lines expressing PML I K/SIMmt or PML I SIMmt were successfully constructed, in which the PML I mutants were overexpressed upon the Dox induction (Figure 4B,C). We examined the degradation of these mutants by either the left or right arm of the bipartite domain. We found that the left arm in virus RHG110 was fully capable of degrading both PML I K/SIMmt and PML I SIMmt (Figure 4D,E, lanes 7–12), whereas the right arm in virus RHG105 was able to degrade PML I SIMmt to an extent similar to that of the wild type ICP0 (Figure 4E, lanes 13–18 and 1–6) but not PML I K/SIMmt (Figure 4D, lanes 13–18). These results suggest that: (i) the right arm functions through a direct recognition of the SUMO moieties on the SUMOylated PML I, regardless of the interactions between the PML I SIM and the SUMO moieties on other ND10 components; and (ii) the left arm uses a mechanism independent of the SUMO-SIM interactions to recognize PML I. Therefore, SIM_362–364_ is the primary substrate recognition mechanism for ICP0, which has the ability to target all SUMOylated PML equally, including both PML isoforms I and II. However, in addition to SIM_362–364_, ICP0 possesses a secondary mechanism, which uses the N-terminal residues 1–83 to specifically single out PML I for a redundant targeting.

### 3.4. The Left Arm of the Bipartite PML I-Interaction Domain Functions When Moved Downstream of the RING Domain

**Figure 5:**
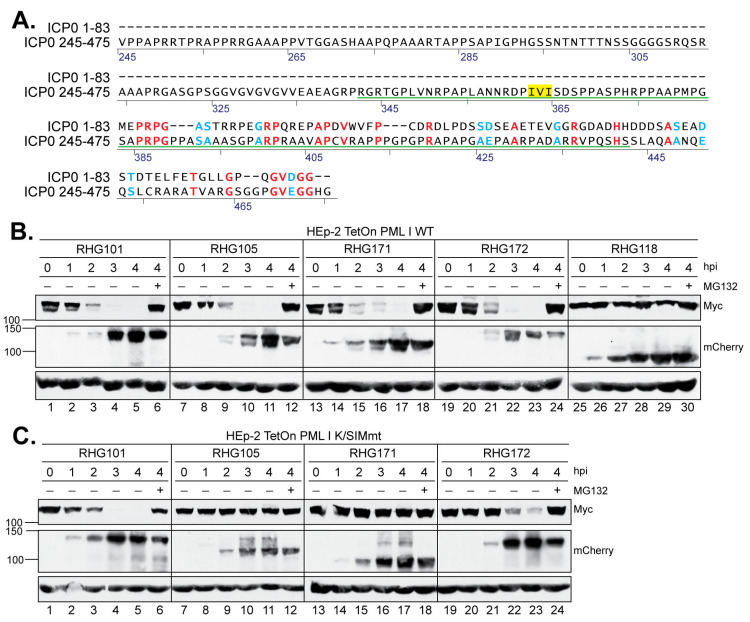
Effects of replacing the homologous sequences in the right arm by the left arm on PML I degradation. (**A**) Sequence alignment of the left and right arms by AlignX from Vector NTI. Identical residues are in red and similar residues are in blue. SIM_362–364_ is highlighted in yellow. (**B**,**C**) HEp-2 TetOn cells expressing PML I WT (**B**) or PML I K/SIMmt (**C**) were induced by Dox overnight and infected by the indicated viruses. At the indicated hpi, infected cell lysates were harvested and probed for the PML I, ICP0, and actin levels as described above.

Sequence analysis showed that the left arm of the bipartite PML I-interaction domain shared modest homology to the second half of the right arm, residues 383–473, with 25% identity and 12% similarity. The homologous region in the right arm substantially overlaps with segment C-D but does not contain a SIM (Figure 5A). To examine whether residues 383–473 play a role in PML I recognition by ICP0, we constructed a double deletion virus RHG171 (Figure 1, group 5), in which the entire left arm and its homologous residues 381–475 in the right arm were both deleted. We found that the double deletion mutant degraded the wild type PML I to the same extent as the full-length ICP0 or ICP0 lacking only the left arm (Figure 5B, lanes 13–18, compared to lanes 1–12). To determine whether this degradation is due to the presence of SIM_362–364_ in RHG171, we performed the degradation time course on HEp-2 TetOn PML I K/SIMmt cells and found that the mutant PML I was not degraded (Figure 5C, lanes 13–18), suggesting that SUMOylation of PML I is required for its recognition by SIM_362–364_ in RHG171. From the results of RHG169 (Figure 3D, lane 2) and RHG171 (Figure 5B), which contain ICP0 residues 245–392 and 245–380, respectively, we conclude that ICP0 SIM_362–364_ is the sole element responsible for the ubiquitination and degradation of SUMOylated PML I carried out by the right arm of the bipartite PML I-interaction domain. However, the small motif SIM_362–364_ must be presented by sufficient surrounding sequences, which have a length requirement but limited sequence stringency. To investigate whether the relative position of the left arm to the RING affects its function, we inserted the left arm sequence downstream of the RING to replace residues 381–475 and constructed a triple mutant ICP0 in the RHG172 virus (Figure 1, group 5). The results showed that the triple mutant had the ability to degrade both the wild type and K/SIMmt form of PML I (Figure 5B,C, lanes 19–24), suggesting that it is the sequence of the left arm, not its location relative to the RING domain, that mediates the secondary recognition of PML I by ICP0.

### 3.5. Both Arms of the Bipartite PML I-Interaction Domain Add Heterologous Polyubiquitin Chains to PML I

**Figure 6:**
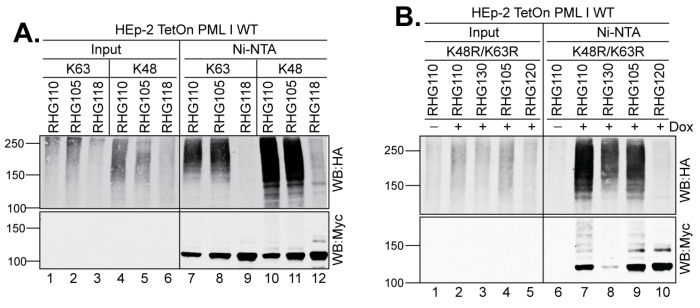
Both arms deposit heterologous polyubiquitin chains to PML I. HEp-2 TetOn PML I WT cells were transfected with the HA-ubiquitin mutated of all lysine residues but K48 or K63 (**A**) or HA-ubiquitin containing the K48R/K63R mutations (**B**), and induced for 24 h before infection with the indicated viruses. After 3 h of infection, cell lysates were subjected to His-tag pulldown to detect the PML I ubiquitination by anti-HA and anti-Myc antibodies as described above.

Ubiquitin has seven lysine residues and each can serve as a substrate for further ubiquitination reactions [2,3,4]. To test whether the two distinctive PML I recognition mechanisms catalyze different ubiquitin linkages in PML I ubiquitination, we transfected the HEp-2 TetOn cells expressing PML I with the HA-tagged ubiquitin constructs that contain mutations in all but one lysine residue. We found that the two most used ubiquitin lysines, K48 and K63, are equally incorporated into the polyubiquitin chain of PML I by either the left or right arm of the bipartite PML I-interaction domain (Figure 6A, lanes 7–8 and 10–11).

We further examined whether ubiquitin lysine residues other than K48 and K63 are involved in the formation of the PML I polyubiquitin chain. For that purpose, we transfected the HEp-2 TetOn cells expressing PML I with an HA-tagged ubiquitin that contains the K48R/K63R substitutions but maintains the intact K6, K11, K27, K29, and K33 residues. Pulldown of the HisMyc-PML I showed that the K48R/K63R ubiquitin mutant was incorporated into the polyubiquitin chain of PML I by either arm (Figure 6B, lanes 7 and 9). Interestingly, in cells infected by the RHG130 virus, in which ICP0 contains only the I362G/V363A/I364G substitutions at SIM_362–364_, the K48R/K63R ubiquitin mutant was obviously incorporated in the PML I polyubiquitin chain but with less intensity compared to that of the ICP0 lacking the entire right arm (Figure 6B, lane 8). Western blotting by the anti-Myc antibody showed much less full-length PML I in the RHG130 infection, demonstrating a more effective degradation of PML I by the RHG130 virus than the RHG110 virus. Likely, there are additional elements in the right arm other than SIM_362–364_ that participate in the regulation of ICP0 E3 activity.

### 3.6. PML I Mutant Not Localized at ND10 Is Degraded via the Left Arm of the Bipartite PML I-Interaction Domain

**Figure 7:**
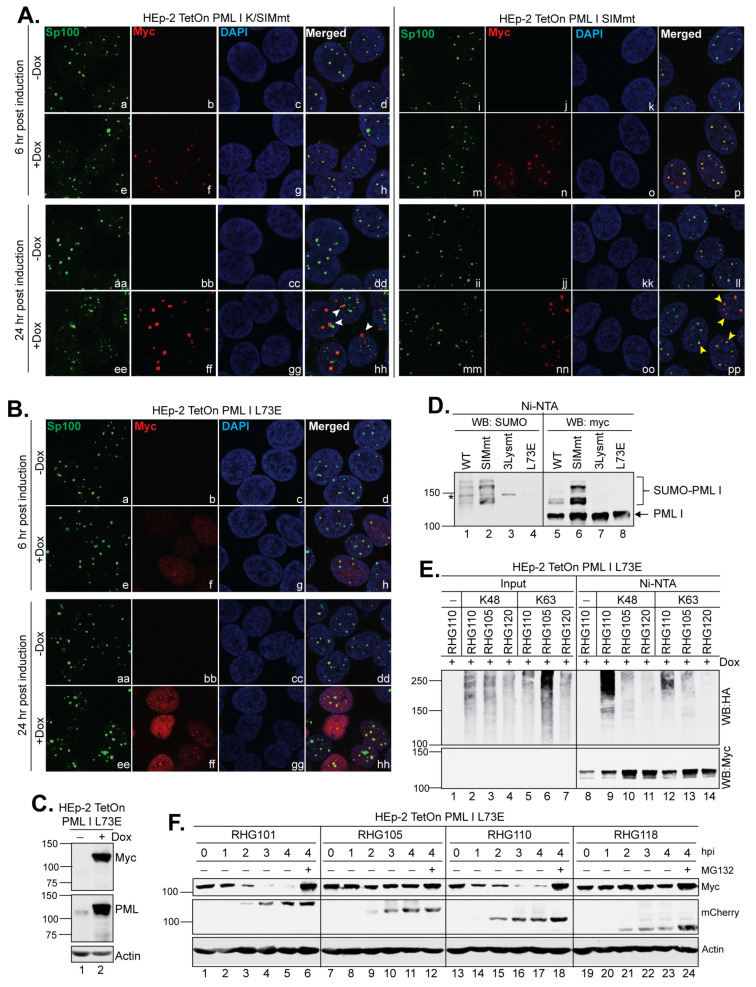
The PML I mutant not localized at ND10 is targeted by the left arm of the bipartite PML I-interaction domain. (**A**,**B**) Subnuclear distribution of the K/SIMmt (**A**), SIMmt (**A**) and L73E (**B**) mutants of PML I. Cells grown in 4-well slides were mock induced or induced by Dox for 6 and 24 h and then fixed, permeabilized, and interacted with the polyclonal anti-Sp100 and monoclonal anti-Myc antibodies. The cells were then reacted with FITC-conjugated goat anti-rabbit and Texas Red-conjugated goat anti-mouse antibodies to be imaged by a Leica TCS SP8 confocal microscope. (**C**) Expression level of PML I L73E detected by Western blotting in the absence or presence of Dox. (**D**) HEp-2 TetOn cells expressing wild type or mutant PML I were induced by Dox overnight and then subjected to His-tag pulldown. The precipitates of wild type or mutant PML I were probed by anti-SUMO and anti-Myc antibodies on Western blots to detect the SUMOylation of PML I. (**E**) HEp-2 TetOn PML I L73E cells were transfected with the HA-ubiquitin mutated of all lysine residues but K48 or K63 and induced for 24 h. Cells were then infected by the indicated viruses and subjected to an in vivo ubiquitination assay as described above. (**F**) HEp-2 TetOn PML I L73E cells were induced overnight and then infected by the indicated viruses. At the indicated hpi, infected cell lysates were harvested and protein levels of PML I L73E mutant, ICP0, and actin were probed by the anti-Myc, anti-mCherry, and acti-actin antibodies as described above.

We examined the subcellular localization of the PML I K/SIMmt and PML I SIMmt mutants. We found that at 6 h post induction, both the newly synthesized mutants were colocalized at ND10 marked with the endogenous Sp100 (Figure 7A, panels h and p), regardless of their SUMOylation status. Both the K/SIMmt and SIMmt mutants of PML I have intact RBCC domains and thereby maintain the ability to interact with endogenous PML isoforms via self-oligomerization [37,42], which recruits PML I K/SIMmt and PML I SIMmt to ND10. In contrast, after 24 h of induction, most PML I K/SIMmt dots were no longer colocalized with detectable Sp100 (Figure 7A, panels hh, white arrowheads), whereas the overexpressed PML I SIMmt was still colocalized with Sp100 (Figure 7A, panels pp, yellow arrowheads). Moreover, with the PML I K/SIMmt overexpression, endogenous ND10 nuclear bodies represented by Sp100 appeared to be disseminated into smaller and less rounded dots compared to that without the PML I K/SIMmt expression (Figure 7B, panels aa and ee), suggesting a severe impact of PML I K/SIMmt on the integrity of the ND10 structure over time.

ND10 nuclear bodies are dynamic nuclear structures involved in many cellular functions, including gene regulation, cell cycle regulation, and anti-viral responses (7–10). PML is the main organizer of ND10 that sustains the ND10 structure and recruits ND10 clients via SUMO-SIM interactions [7]. Results from Figure 4D and Figure 7A indicate that the left arm of the bipartite PML I-interaction domain in the RHG110 virus has the ability to degrade PML I K/SIMmt without the regular interactions with other ND10 components. To confirm that the left arm is capable of recognizing unSUMOylated PML I outside the context of ND10, we constructed another PML I mutant that contained an L73E substitution in the RBCC domain, which is known to disrupt the self-oligomerization of PML [42]. We found that the expression of PML I L73E was tightly regulated by the Dox induction (Figure 7C), and this mutant was diffused throughout the nucleus at both 6 and 24 h post induction (Figure 7B, panels f and ff). Consistent with a previous report [42], the failure in self-oligomerization of PML I L73E prevented it from being SUMOylated, similar to the PML I 3Lysmt that contained the K65R/K160R/K490R substitutions, despite the presence of SUMOylation sites in the L73E mutant (Figure 7D, lanes 3–4 and 7–8). Serving as controls, both the wild type PML I and PML I SIMmt were clearly SUMOylated (Figure 7D, lanes 1–2 and 5–6). The results showed that PML I L73E, although diffused away from ND10 and not SUMOylated, was successfully polyubiquitinated with either K48 or K63 of ubiquitin (Figure 7E, lanes 9 and 12) and degraded within 4 h after infection (Figure 7F, lanes 13–18) by the left arm of RHG110 virus. On the contrary, the ICP0 containing only the right arm in the RHG105 virus failed to either ubiquitinate or degrade the unSUMOylated PMLI (Figure 7E, lanes 10 and 13, and Figure 7F, lanes 7–12), demonstrating a differentiated recognition of PML I by the left or right arm of the bipartite PML I-interaction domain.

### 3.7. The Left Arm of Bipartite PML I-Interaction Domain Is Required for PML I to Be Degraded in the Cytoplasm in Late Infection

**Figure 8:**
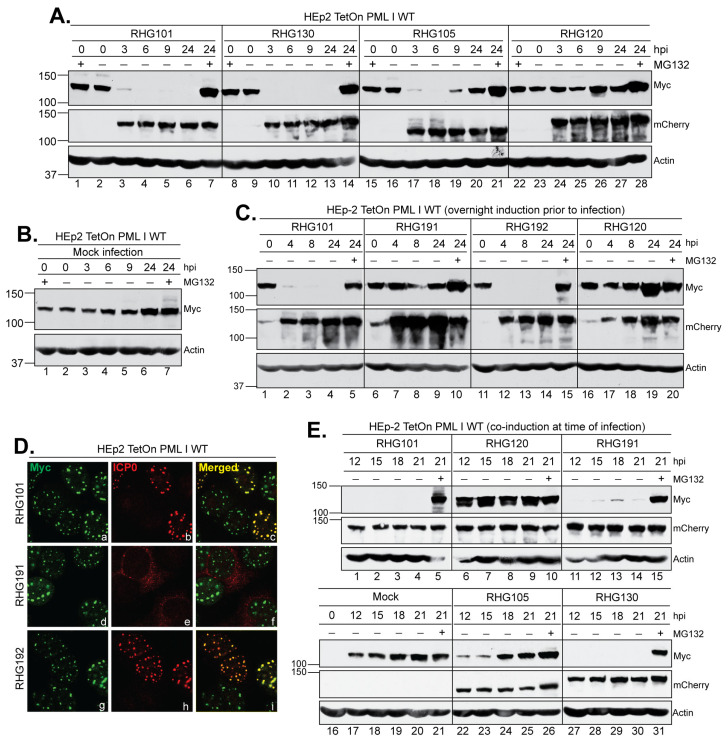
The left arm of the bipartite PML I-interaction domain is required for targeting PML I in the cytoplasm. (**A**–**C**) HEp-2 TetOn PML I WT cells were induced overnight and then mock infected (**B**) or infected by the indicated viruses (**A**,**C**) in the absence or presence of MG132. Infected cell lysates were collected throughout the course of infection to probe for PML I, ICP0, and actin as described above. (**D**) HEp-2 TetOn PML I WT cells were induced overnight and then infected by the indicated viruses for 2 h before being fixed, permeabilized, and reacted with polyclonal anti-ICP0 and monoclonal anti-Myc antibodies. The cells were then reacted with Alexa 594-conjugated goat anti-rabbit and Alexa 488-conjugated goat anti-mouse antibodies to show the subcellular distribution of ICP0 relative to the HisMyc-tagged PML I. (**E**) HEp-2 TetOn PML I WT cells were co-induced at the time of infection by the indicated viruses. Infected cell lysates were collected at a late hpi to probe for PML I, ICP0, and actin as described above.

The right arm of the bipartite domain uses SIM_362–364_ to target PML I, the same mechanism as in its targeting of the PML isoforms II, IV, and VI. Most likely, the SIM_362–364_-dependent substrate recognition is the major path for ICP0 to target its substrates, whereas the left arm acts as a secondary mechanism to enhance the degradation of unSUMOylated PML I, but not the rest of the PML isoforms. An important question lingering is why HSV-1 specifically singles out PML I for a redundant degradation. To fully understand the role of PML I in HSV-1 infection, we examined the PML I level in the entire infection cycle. We found that after an overnight induction, the wild type PML I level remained consistent in mock infected cells, with only a slight increase at 24 h and no substantial accumulation in the presence of MG132 (Figure 8B), suggesting that the ectopic PML I is fairly stable without the HSV-1 infection. Between 3 to 6 h after HSV-1 infection, PML I was degraded by the wild type ICP0, ICP0 mutated at SIM_362–364_, or ICP0 lacking the left arm, but remained stable when the RING-type E3 was inactivated in RHG120-infected cells (Figure 8A). Interestingly, in cells infected by the RHG105 that lacks the left arm, the PML I level increased again between 9 and 24 hpi (Figure 8A, lanes 19–20). The reappearance of PML I was not observed in infections by the RHG101 or RHG130 virus, suggesting that the ICP0 lacking the left arm fails to enforce the PML I degradation late in HSV-1 infection.

During HSV-1 infection, ICP0 undergoes subcellular trafficking at different infection phases. Early in infection, nascent ICP0 is immediately localized to ND10 upon its synthesis. While at ND10, ICP0 targets PML and Sp100 for proteasomal degradation, causing the dispersal of ND10 components as well as ICP0 itself. Later on, ICP0 is translocated to the cytoplasm after the onset of DNA replication [25,43]. To examine whether the left arm function in late infection is associated with the cytoplasmic ICP0, we first constructed a mutant ICP0 lacking NLS and examined its E3 ubiquitin ligase activity. We found that after an overnight induction, infection by the RHG191 virus, which contains the R501Q/R503A/K504Q/R505A/R506Q substitutions in ICP0 NLS (NLSmt), did not cause PML I to degrade either at the early or late infection phase (Figure 8C, lanes 6–10). Likely, the inability of ICP0 NLSmt to enter the nucleus in the RHG191 infection (Figure 8D, panel e) restrained its access to the nuclear PML I expressed prior to infection. When we inserted the SV40 NLS at ICP0 residues 501–506 in the RHG192 virus, we found that the reconstitution of nuclear localization (Figure 8D, panel h) restored PML I degradation (Figure 8C, lanes 11–15), suggesting that: (i) the basic amino acids in the conventional NLS are solely responsible for the nuclear import of ICP0, and (ii) the nuclear localization of ICP0 is essential for ICP0 to approach and target ND10 in early infection. To test whether ICP0 NLSmt has the ability to degrade PML I in the cytoplasm, we co-induced PML I at the time of infection so ICP0 NLSmt was present in the cytoplasm and ready to act when the PML I was being synthesized. Consistent with what we observed in the pre-induction experiments (Figure 8B), the co-induced PML I remained stable throughout the time course in mock infected cells (Figure 8E, lanes 16–21). Compared to the RHG101 and RHG120 viruses, which serve as the positive and negative controls of the ICP0 E3 ubiquitin ligase activity, co-induction at the time of infection did not lead to PML I accumulation at 12 to 21 h in RHG191 infected cells, suggesting that PML I degradation occurred in the cytoplasm by ICP0 NLSmt (Figure 8E, lanes 11–15). We further examined the role of left arm or SIM_362–364_ of the right arm in the cytoplasmic degradation of PML I. We found that while mSIM_362–364_ of the RHG130 virus did not affect the degradation of ICP0 in the cytoplasm, the lack of the left arm in the RHG105 virus caused PML I to accumulate at 12 to 21 hpi, indicating the requirement of the left arm in the cytoplasmic degradation of PML I late in HSV-1 infection.

### 3.8. The Secondary Degradation of PML I Triggered by the Left Arm Prevents the Reformation of ND10 in Late Infection

**Figure 9:**
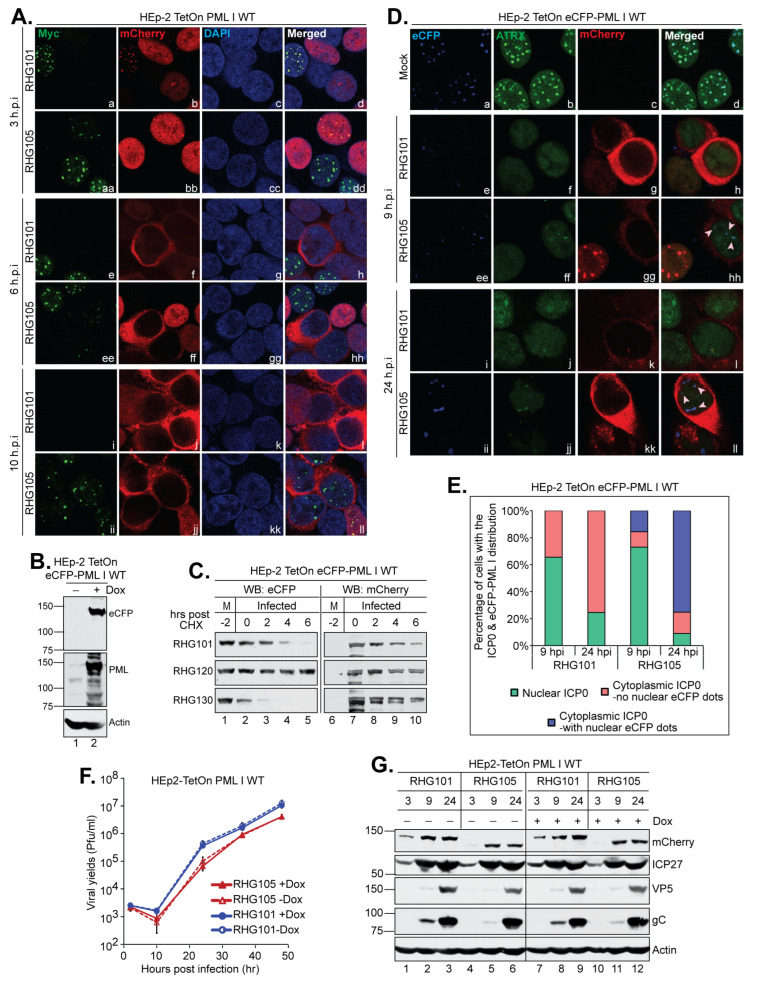
The left arm of the bipartite PML I-interaction domain is required to reform ND10-like structures late in HSV-1 infection. (**A**) HEp-2 TetOn PML I WT cells seeded on 4-well slides were induced overnight and then infected by the indicated viruses. At the indicated hpi, cells were fixed, permeabilized, and reacted with monoclonal anti-Myc and polyclonal anti-mCherry antibodies. The cells were then reacted with Alexa 488-cojugated goat anti-mouse and Alexa 594-conjugated goat anti-rabbit antibodies to be imaged by a confocal microscope. (**B**) Expression of an eCFP-tagged wild type PML I in HEp-2 TetOn cells in the absence and presence of Dox. (**C**) HEp-2 TetOn eCFP PML I cells were induced overnight and then infected by 10 pfu/cell of the indicated viruses. After 2 h of infection, CHX was added and a time course was taken to examine the half-life of eCFP-PML I and ICP0 by the anti-eCFP and anti-mCherry antibodies via Western blotting. (**D**) HEp-2 TetOn eCFP-PML I WT cells were induced by Dox and then mock infected or infected by the indicated viruses for 9 and 24 h. Cells were reacted with polyclonal anti-ATRX and monoclonal anti-mCherry antibodies and then stained by FITC-conjugated goat anti-rabbit and Texas Red-conjugated goat anti-mouse antibodies to be imaged by a Leica TCS SP8 confocal microscope. (**E**) Cells from (**D**) were tabulated by the distribution of ICP0 and eCFP-PML I and plotted with Microsoft Excel. (**F**) HEp-2 TetOn PML I WT cells were mock or Dox induced and then infected by the indicated viruses at 0.05 pfu/cell. At 2, 10, 24, 36 and 48 hpi, infected cell lysates were titrated on U2OS cells to generate viral growth curves. (**G**) HEp-2TetOn PML I WT cells were mock or Dox induced and then infected by RHG101 or RHG105 at 10 pfu/cell. The total infected cell lysates were collected at the indicated times. Viral proteins were probed with the indicated antibodies by Western blotting.

Next, we inspected the subcellular distribution of PML I in late infection. Consistent with previous observations [22,40], both the mCherry-tagged wild type ICP0 and ICP0 lacking a left arm followed the regular route of trafficking, from ND10 to filling the nucleus and then to solely in the cytoplasm (Figure 9A, the mCherry panels) [43]. At 3 and 6 hpi, the loss of PML I in the infected cells was obvious in both RHG101 and RHG105 infected cells. However, at 10 hpi, PML I reappeared as dotted structures in the nucleus of the RHG105-infected cells when ICP0 was solely in the cytoplasm, whereas in the RHG101-infected cells with ICP0 in the cytoplasm, PML I was not present (Figure 9A, panels i–l and ii–ll). To examine whether the PML I nuclear dots reformed in late infection were relevant to ND10, we constructed a HEp-2 TetOn stable cell line that expressed an eCFP-tagged PML I (Figure 9B). Same as the HisMyc-PML I (34), the eCFP-PML I was degraded by ICP0 in a SIM_362–364_-independent manner (Figure 9C). We then examined ATRX, an ND10 constituent that is not degraded but is dispersed upon PML degradation, to observe ATRX localization relative to eCFP-PML I in late infection (Figure 9D). We tabulated over a hundred infected cells at 9 and 24 hpi to analyze the PML I dots. We found that at 10 pfu/cell, infections of both RHG101 and RHG105 progress similarly, with 65.5% and 73.0% of infected cells having a nuclear ICP0 at 9 hpi and 75.5% and 91.0% of infected cells having a cytoplasmic ICP0 at 24 hpi, respectively. None of the cytoplasmic ICP0-containing cells in the RHG101 infection had residual eCFP signals at either 9 or 24 hpi. However, in the RHG105 infection, 15.6% and 75.2% of cells with the cytoplasmic ICP0 had dotted eCFP signals in nuclei, at 9 and 24 hpi, respectively (Figure 9E). More importantly, the eCFP-PML I dots were colocalized with ATRX in the nucleus, indicating the reformation of ND10-like structures late in the RHG105 infection (Figure 9D, pink arrowheads). The fact that more RHG105-infected cells contained dotted eCFP-PML I at 24 hpi than those of 9 hpi strongly suggests that these dots are not due to an incomplete dispersal of ND10 at early infection, but rather a reformation of ND10 at late infection. Therefore, we conclude that the left arm of the bipartite PML I-interaction domain enforces a continuous PML I degradation to prevent the reformation of ND10 throughout the infection cycle.

We further examined the effects of the ICP0 N-terminus on the HSV-1 replication by comparing the viral growth and protein expression of the RHG101 and RHG105 viruses. We found that deletion of the ICP0 N-terminus caused a moderate reduction in viral yields, reaching to 6.6 × 10^4^ and 1.0 × 10^5^ pfu/mL at 24 hpi and 4.1 × 10^6^ and 4.1 × 10^6^ pfu/mL at 48 hpi in the presence and absence of PML I expression, respectively. In comparison, the wild type ICP0 containing RHG101 yields the titer of 3.6 × 10^5^ and 4.4 × 10^5^ pfu/mL at 24 hpi and 1.0 × 10^7^ and 1.3 × 10^7^ pfu/mL at 48 hpi, in the presence and absence of PML I expression, respectively (Figure 9F). A delay of expression of the true late protein, gC, was also observed regardless of PML I overexpression in RHG105-infected cells compared to that of RHG101, whereas ICP27 (immediate early) and VP5 (leaky late) protein levels remain comparable throughout the two infections (Figure 9G), suggesting a mild delay in viral production.

## 4. Discussion

ICP0 of HSV-1 is a multifunctional viral protein key to the establishment of viral replication. It has E3 ubiquitin ligase activity in the RING finger domain and several interactive domains scattered throughout the sequence [44]. By manipulating its E3 substrates and interaction partners, ICP0 modulates pathways such as interferon responses, the ubiquitin-proteasome system, chromatin remodeling pathway, and DNA damage responses to counteract various anti-viral defenses and orchestrate an effective infection [26,45]. How ICP0 spatially and temporally coordinates these counteractions to facilitate viral replication, however, is not well understood. We previously reported that disruption of the ICP0–CoREST interaction affects the degradation of PML by ICP0 [30], indicating the coordination between the RING-type E3 and other interactive domains of ICP0. Potentially, a spatiotemporally regulated interaction network of ICP0 affecting the activity and specificity of ICP0 E3 empowers the virus to simultaneously control the host defenses on multiple fronts. We seek to delineate ICP0 elements regulating the E3 domain as the first step towards understanding the complex role of ICP0 multifunctionality in HSV-1 infection and pathogenesis.

ICP0 has a strict specificity for substrate recognition. It differentiates similar substrates such as PML isoforms, which differ only in their C-termini, and targets them in a SIM_362–364_-dependent or -independent manner [36]. The present study focuses on identifying the ICP0 elements that regulate the SIM_362–364_-independent degradation of PML isoform I to understand the role of ICP0 E3 specificity in HSV-1 infection. We conducted mutagenesis studies with both ICP0 and PML I to characterize the regulatory effects of the left or right arm of the bipartite PML I-interaction domain on substrate recognition of ICP0. We found that the right arm of the bipartite domain uses SIM_362–364_ to degrade SUMOylated PML I, just like how it does with several other PML isoforms. Previously, we showed that the PML II isoform containing the K/SIMmt mutant was recruited to ND10 and interfered with the degradation of endogenous PML and Sp100 in HSV-1 infection [37], suggesting that SUMOylated ICP0 substrates located at ND10 are accessed as a whole through the SUMO-interaction network within ND10. Here, we showed that length-dependent support from sequences surrounding SIM_362–364_ facilitated the SIM_362–364_-mediated ubiquitination and degradation of PML I (Figure 3), suggesting the presence of additional sequence-irrelevant elements in the regulation of SUMO-SIM based substrate recognition of ICP0 E3. The ND10 structural formation involves liquid–liquid phase separation in its formation, a process that allows the demixing of diffusible molecules into separated phases so that the molecule concentration within the phase boundary is highly elevated for certain activities [7,46,47]. Further investigation of the role of phase separation in the SIM_362–364_-dependent ICP0 substrate recognition will be critical to the understanding of both the activity and specificity of the ICP0 E3.

In addition to the primary recognition, ICP0 applies a secondary recognition mechanism to PML I, which depends on ICP0 residues 1–83 to carry out a redundant degradation of PML I outside ND10 or in the cytoplasm regardless of its SUMOylation status (Figure 7 and Figure 8). This is the first report to identify and characterize a supplementary substrate recognition of ICP0 that specifically targets PML I. The key is why HSV-1 needs to single out PML I for a redundant degradation. Increasing evidence has shown that PML isoforms use their specific C-terminus to interact with different proteins and carry out isoform-specific functions in gene regulation, tumor suppression, and virus infection [48,49]. Likely, the redundancy in PML I substrate recognition, which does not apply to PML II, IV, and VI [36], is associated with the special functions of PML I in HSV-1 replication. The unique sequences in the C-terminus of PML I contains a nuclear export signal (NES) that allows PML I to shuttle between the nucleus and cytoplasm [50]. Interestingly, both the PML I overexpression and inhibition of nuclear export promote the reformation of ND10 in cells derived from acute promyelocytic leukemia [51], suggesting that a high level of PML I in the nucleus may contribute to the formation of ND10. We demonstrated that in RHG105-infected cells, the lack of secondary degradation of PML I leads to a resurgence of PML I and reformation of ND10-like structures late in HSV-1 infection, while ICP0 has been translocated into the cytoplasm after the DNA replication [43]. Presumably, the potential that PML I leaves ND10 and shuttles between the nucleus and cytoplasm provides an opportunity for PML I to escape if ICP0 only targets it at ND10. The secondary mechanism by ICP0 ensures a complete wipeout of PML I, at ND10, outside ND10, or in the cytoplasm to prevent it from reinitiating ND10 formation after ICP0 translocation into the cytoplasm. One important question to ask next is whether there are other ICP0 substrates using this secondary mechanism. Identification of additional substrates using the same mechanism will help us understand why ICP0 develops such redundancy and how the secondary recognition affects HSV-1 infection.

Both the primary and secondary mechanisms of PML I substrate recognition render ICP0 to use multiple ubiquitin lysine residues to ubiquitinate PML I. The incorporation of K48, K63, and other ubiquitin lysines into the polyubiquitin chain of PML I leads to the same outcome—degradation of PML I. These results imply that a fast and complete degradation of PML I may be advantageous for HSV-1 replication. However, one-step growth curves showed that viral replication of RHG101 and RHG105 is not affected by the overexpression of PML I, suggesting that ICP0 SIM_362–364_ itself has an excessive ability to overpower the ectopic PML I at ND10 in early infection. The dynamic and complex nature of ND10 may also offset the PML I overexpression by changing ND10 composition or isoform interactions in the presence of a high level of ectopic PML I, which can dampen the effects of PML I overexpression on the overall viral yield. Again, a careful investigation of ND10 dynamics via phase separation may provide clues for the roles of different PML isoforms in HSV-1 infection.

The loss of the ICP0 N-terminus showed a moderate reduction of viral yield and a delayed expression of the true late protein gC, indicating a role of the ICP0 N-terminus in HSV-1 infection. ND10 has been reported to have both positive and negative effects on HSV-1 and other herpes infections [27,52]. It remains unclear whether the differential PML ubiquitination catalyzed by ICP0 affects the interaction between ICP0 and other ND10 components besides dispersing the ND10 structure and, more importantly, whether there are ND10 components differentially ubiquitinated by ICP0 but which are not degraded via proteasomes. To understand how the ICP0 N-terminus regulates HSV-1 replication via the PML I ubiquitination, a survey of cellular and viral proteins binding at the ICP0 N-terminus and a profile of the composition of ND10-like structures formed late in RHG105 infection are needed for further mechanistic studies.

## Data Availability

Not applicable.
